# Supplementary material for: Deletion of wfs1 Impairs Oligodendrocyte Precursor Cells Dorsal Distribution and Myelination Through the wfs1-hmgcs1 Axis in Zebrafish
Source: Biology (Basel). 2026 Aug 16;15(16):1402. doi: 10.3390/biology15161402 (PMC13510151; doi:10.3390/biology15161402)
Supplement: Supplementary file 1 [file biology-15-01402-s001.zip › File S1-Transcriptome sequencing data and differentially expressed genes (DEGs) in wfs1-- zebrafish at 5 dpf/2.KEGG/wfs1KOvsWT/GSEA/WT/TIGHT_JUNCTION/TIGHT_JUNCTION(DRE04530).html]

Details for gene set TIGHT\_JUNCTION(DRE04530)[GSEA]

|  || Dataset | deg.sample |
| Phenotype | sample.cls |
| Upregulated in class | WT |
| GeneSet | TIGHT\_JUNCTION(DRE04530) |
| Enrichment Score (ES) | -0.23075859 |
| Normalized Enrichment Score (NES) | -1.3369799 |
| Nominal p-value | 0.0 |
| FDR q-value | 0.7946736 |
| FWER p-Value | 0.926 |
Table: GSEA Results Summary

  

Fig 1: Enrichment plot: TIGHT\_JUNCTION(DRE04530)      
 Profile of the Running ES Score & Positions of GeneSet Members on the Rank Ordered List

  

| PROBE | DESCRIPTION (from dataset) | GENE SYMBOL | GENE\_TITLE | RANK IN GENE LIST | RANK METRIC SCORE | RUNNING ES | CORE ENRICHMENT || 1 | 57934\_1 | actb1 |  |  | 1 | 4.982 | 0.0279 | No |
| 2 | 337226\_1 | zgc:153867 |  |  | 121 | 4.641 | 0.0502 | No |
| 3 | 792828\_1 | pard6a |  |  | 627 | 2.729 | 0.0496 | No |
| 4 | 556226\_1 | cdc42l2 |  |  | 680 | 2.623 | 0.0627 | No |
| 5 | 793158\_1 | cdc42l |  |  | 826 | 2.351 | 0.0713 | No |
| 6 | 677750 | myl2b |  |  | 901 | 2.226 | 0.0814 | No |
| 7 | 436718 | rhocb |  |  | 948 | 2.171 | 0.0922 | No |
| 8 | 798632 | myl6 |  |  | 950 | 2.168 | 0.1043 | No |
| 9 | 286739 | msna |  |  | 1004 | 2.088 | 0.1143 | No |
| 10 | 797651\_1 | jam3a |  |  | 1491 | 1.658 | 0.1083 | No |
| 11 | 559392\_1 | ppp2r2cb |  |  | 1654 | 1.567 | 0.1119 | No |
| 12 | 101882911 | LOC101882911 |  |  | 1827 | 1.446 | 0.1146 | No |
| 13 | 793143 | si:dkey-98f17.3 |  |  | 1831 | 1.442 | 0.1226 | No |
| 14 | 100005566 | f11r.2 |  |  | 1968 | 1.381 | 0.1261 | No |
| 15 | 431777 | tuba7l |  |  | 2071 | 1.338 | 0.1303 | No |
| 16 | 373080 | tuba1b |  |  | 2072 | 1.337 | 0.1379 | No |
| 17 | 793952\_1 | si:dkey-253i9.4 |  |  | 2841 | 1.073 | 0.1196 | No |
| 18 | 101884483 | LOC101884483 |  |  | 2886 | 1.061 | 0.1241 | No |
| 19 | 368619\_1 | cldn10a |  |  | 2912 | 1.057 | 0.1293 | No |
| 20 | 101884950 | amotl1 |  |  | 2934 | 1.054 | 0.1345 | No |
| 21 | 100333863 | LOC100333863 |  |  | 2957 | 1.048 | 0.1397 | No |
| 22 | 81581 | cldnb |  |  | 3068 | 1.021 | 0.1420 | No |
| 23 | 573216 | tuba1a |  |  | 3240 | 0.984 | 0.1421 | No |
| 24 | 563016 | zgc:154006 |  |  | 3277 | 0.977 | 0.1464 | No |
| 25 | 406820 | actr2a |  |  | 3314 | 0.969 | 0.1507 | No |
| 26 | 561276 | arhgef2 |  |  | 3329 | 0.967 | 0.1557 | No |
| 27 | 406582 | ppp2cb |  |  | 3436 | 0.944 | 0.1577 | No |
| 28 | 571897 | rab8a |  |  | 3734 | 0.882 | 0.1532 | No |
| 29 | 406559 | cldn5a |  |  | 3846 | 0.860 | 0.1545 | No |
| 30 | 337223 | tuba8l |  |  | 3910 | 0.846 | 0.1573 | No |
| 31 | 403311 | cacna1db |  |  | 4082 | 0.814 | 0.1564 | No |
| 32 | 101883343 | LOC101883343 |  |  | 4104 | 0.810 | 0.1603 | No |
| 33 | 678612 | zgc:136892 |  |  | 4271 | 0.781 | 0.1595 | No |
| 34 | 100093706\_2 | rab8b |  |  | 4563 | 0.737 | 0.1544 | No |
| 35 | 100001136 | si:ch73-61d6.3 |  |  | 4626 | 0.732 | 0.1565 | No |
| 36 | 572074 | prkaa2 |  |  | 4710 | 0.717 | 0.1579 | No |
| 37 | 567620 | si:ch211-95j8.5 |  |  | 4730 | 0.714 | 0.1613 | No |
| 38 | 793648 | LOC793648 |  |  | 4799 | 0.705 | 0.1631 | No |
| 39 | 751630 | zgc:153713 |  |  | 4826 | 0.702 | 0.1663 | No |
| 40 | 100001198 | prkaa1 |  |  | 5040 | 0.675 | 0.1633 | No |
| 41 | 100093706 | rab8b |  |  | 5433 | 0.638 | 0.1545 | No |
| 42 | 100124652 | pard6b |  |  | 5488 | 0.635 | 0.1563 | No |
| 43 | 81590 | cldn1 |  |  | 5644 | 0.622 | 0.1549 | No |
| 44 | 560912 | si:dkey-92j12.5 |  |  | 6013 | 0.585 | 0.1466 | No |
| 45 | 561211 | igsf5a |  |  | 6060 | 0.581 | 0.1484 | No |
| 46 | 393998 | prkacba |  |  | 6234 | 0.564 | 0.1461 | No |
| 47 | 678556 | cldn15b |  |  | 6249 | 0.562 | 0.1488 | No |
| 48 | 101884425 | LOC101884425 |  |  | 6612 | 0.527 | 0.1403 | No |
| 49 | 325084 | src |  |  | 6875 | 0.502 | 0.1348 | No |
| 50 | 100332055 | prkceb |  |  | 6982 | 0.493 | 0.1342 | No |
| 51 | 65236 | mapk8b |  |  | 6998 | 0.492 | 0.1365 | No |
| 52 | 334086\_2 | hspa4b |  |  | 7029 | 0.489 | 0.1383 | No |
| 53 | 792024 | si:ch73-199e17.1 |  |  | 7117 | 0.481 | 0.1382 | No |
| 54 | 100005496 | si:ch211-1i11.3 |  |  | 7276 | 0.468 | 0.1359 | No |
| 55 | 393154 | tuba8l4 |  |  | 7289 | 0.467 | 0.1381 | No |
| 56 | 335916 | jun |  |  | 7293 | 0.467 | 0.1406 | No |
| 57 | 436612\_2 | cldn7a |  |  | 7392 | 0.461 | 0.1401 | No |
| 58 | 100093706\_1 | rab8b |  |  | 7432 | 0.458 | 0.1415 | No |
| 59 | 561423 | LOC561423 |  |  | 7472 | 0.455 | 0.1428 | No |
| 60 | 80959 | pard6gb |  |  | 7495 | 0.454 | 0.1446 | No |
| 61 | 103909610 | LOC103909610 |  |  | 7518 | 0.453 | 0.1465 | No |
| 62 | 445278 | cldn8 |  |  | 7631 | 0.444 | 0.1454 | No |
| 63 | 252845 | mpp5a |  |  | 7948 | 0.428 | 0.1378 | No |
| 64 | 100334365 | LOC100334365 |  |  | 7976 | 0.425 | 0.1394 | No |
| 65 | 100006132 | si:ch211-1f22.2 |  |  | 8076 | 0.417 | 0.1386 | No |
| 66 | 336504 | arpc5a |  |  | 8597 | 0.395 | 0.1243 | No |
| 67 | 445070 | cldnk |  |  | 8659 | 0.390 | 0.1246 | No |
| 68 | 110439241 | LOC110439241 |  |  | 8714 | 0.387 | 0.1251 | No |
| 69 | 100005496\_1 | si:ch211-1i11.3 |  |  | 8818 | 0.380 | 0.1239 | No |
| 70 | 100005301\_1 | jam2b |  |  | 8820 | 0.380 | 0.1260 | No |
| 71 | 393501 | marveld3 |  |  | 8946 | 0.371 | 0.1242 | No |
| 72 | 560913 | map2k7 |  |  | 9023 | 0.366 | 0.1238 | No |
| 73 | 100147871 | LOC100147871 |  |  | 9032 | 0.365 | 0.1256 | No |
| 74 | 569084 | cldni |  |  | 9262 | 0.349 | 0.1203 | No |
| 75 | 405757 | oclna |  |  | 9311 | 0.346 | 0.1207 | No |
| 76 | 568833 | cldn34a |  |  | 9439 | 0.338 | 0.1186 | No |
| 77 | 553319 | prkag3a |  |  | 9526 | 0.332 | 0.1177 | No |
| 78 | 407987 | arhgef18a |  |  | 9553 | 0.330 | 0.1188 | No |
| 79 | 368723 | inadl |  |  | 9577 | 0.328 | 0.1199 | No |
| 80 | 566060 | tjap1 |  |  | 9697 | 0.321 | 0.1179 | No |
| 81 | 335158 | wasb |  |  | 9730 | 0.319 | 0.1187 | No |
| 82 | 393664 | tuba8l2 |  |  | 9767 | 0.318 | 0.1194 | No |
| 83 | 555805 | vaspa |  |  | 9786 | 0.317 | 0.1206 | No |
| 84 | 100329779 | rock1 |  |  | 9828 | 0.314 | 0.1210 | No |
| 85 | 101886267 | cldn20 |  |  | 9864 | 0.312 | 0.1217 | No |
| 86 | 562720 | mapk8a |  |  | 9888 | 0.310 | 0.1227 | No |
| 87 | 378845 | tjp1a |  |  | 9932 | 0.308 | 0.1231 | No |
| 88 | 795670 | llgl2 |  |  | 9936 | 0.307 | 0.1247 | No |
| 89 | 327377 | tuba4l |  |  | 9990 | 0.304 | 0.1247 | No |
| 90 | 403009 | ezra |  |  | 10033 | 0.301 | 0.1251 | No |
| 91 | 100004456 | cldn10b |  |  | 10152 | 0.293 | 0.1230 | No |
| 92 | 560400 | actn1 |  |  | 10299 | 0.285 | 0.1200 | No |
| 93 | 570761 | prkag2a |  |  | 10629 | 0.265 | 0.1110 | No |
| 94 | 405816 | ppp2r2d |  |  | 10706 | 0.260 | 0.1101 | No |
| 95 | 373089 | tjp3 |  |  | 10740 | 0.259 | 0.1105 | No |
| 96 | 563421 | amot |  |  | 10806 | 0.255 | 0.1099 | No |
| 97 | 114446\_1 | dlg1 |  |  | 10908 | 0.249 | 0.1081 | No |
| 98 | 393550 | arpc5la |  |  | 10926 | 0.248 | 0.1089 | No |
| 99 | 450023 | cldn5b |  |  | 11090 | 0.239 | 0.1051 | No |
| 100 | novel.843 | - |  |  | 11116 | 0.238 | 0.1057 | No |
| 101 | 368473 | scrib |  |  | 11255 | 0.230 | 0.1026 | No |
| 102 | 558810 | ppp2r2bb |  |  | 11322 | 0.226 | 0.1018 | No |
| 103 | 558920 | amotl2a |  |  | 11469 | 0.218 | 0.0984 | No |
| 104 | 550487 | vaspb |  |  | 11562 | 0.213 | 0.0966 | No |
| 105 | 100537244 | myl2a |  |  | 11772 | 0.202 | 0.0912 | No |
| 106 | 565459 | tjp1b |  |  | 11806 | 0.201 | 0.0912 | No |
| 107 | 497648 | dlg1l |  |  | 11820 | 0.200 | 0.0920 | No |
| 108 | 322221 | actn4 |  |  | 11832 | 0.199 | 0.0927 | No |
| 109 | 556104 | arpc5lb |  |  | 11981 | 0.192 | 0.0891 | No |
| 110 | 405887 | nf2b |  |  | 11996 | 0.192 | 0.0898 | No |
| 111 | 550468 | zgc:112437 |  |  | 12137 | 0.183 | 0.0864 | No |
| 112 | 335865 | hspa4a |  |  | 12222 | 0.178 | 0.0847 | No |
| 113 | 317747 | rock2a |  |  | 12313 | 0.173 | 0.0828 | No |
| 114 | 110438074 | whamm |  |  | 12419 | 0.168 | 0.0804 | No |
| 115 | 724017 | crb3a |  |  | 12508 | 0.164 | 0.0786 | No |
| 116 | 100535324\_1 | mpp4a |  |  | 12657 | 0.156 | 0.0748 | No |
| 117 | 406582\_1 | ppp2cb |  |  | 12771 | 0.151 | 0.0720 | No |
| 118 | 327272 | slc9a3r1a |  |  | 12919 | 0.145 | 0.0682 | No |
| 119 | 100332627 | epb41l4b |  |  | 13036 | 0.138 | 0.0653 | No |
| 120 | 493612 | zgc:101810 |  |  | 13049 | 0.137 | 0.0657 | No |
| 121 | 570216 | itgb1b |  |  | 13091 | 0.136 | 0.0652 | No |
| 122 | 335032 | rapgef6 |  |  | 13144 | 0.133 | 0.0643 | No |
| 123 | 81591 | cldn15la |  |  | 13243 | 0.129 | 0.0619 | No |
| 124 | 368266 | arhgef1a |  |  | 13321 | 0.124 | 0.0601 | No |
| 125 | 558752 | ppp2r2aa |  |  | 13396 | 0.121 | 0.0585 | No |
| 126 | 796314 | LOC796314 |  |  | 13479 | 0.117 | 0.0565 | No |
| 127 | 394243 | arpc1a |  |  | 13546 | 0.113 | 0.0551 | No |
| 128 | 334086\_1 | hspa4b |  |  | 13577 | 0.112 | 0.0548 | No |
| 129 | 445305\_2 | arpc4l |  |  | 13646 | 0.109 | 0.0532 | No |
| 130 | 100329719 | slc9a3r1b |  |  | 13769 | 0.103 | 0.0499 | No |
| 131 | 569698 | mapk10 |  |  | 13907 | 0.096 | 0.0461 | No |
| 132 | 563147 | prkacbb |  |  | 13940 | 0.095 | 0.0457 | No |
| 133 | 335474 | arpc1b |  |  | 13993 | 0.093 | 0.0445 | No |
| 134 | 559634 | nedd4l |  |  | 14142 | 0.085 | 0.0403 | No |
| 135 | 100331226 | si:ch73-272h16.4 |  |  | 14201 | 0.082 | 0.0390 | No |
| 136 | 30483 | gata4 |  |  | 14212 | 0.082 | 0.0391 | No |
| 137 | 550585 | ppp2ca |  |  | 14235 | 0.081 | 0.0389 | No |
| 138 | 100170824 | pard6ga |  |  | 14339 | 0.077 | 0.0360 | No |
| 139 | 558359 | mllt4a |  |  | 14445 | 0.072 | 0.0331 | No |
| 140 | 415107 | bves |  |  | 14449 | 0.072 | 0.0334 | No |
| 141 | 564064 | prkacaa |  |  | 14469 | 0.071 | 0.0332 | No |
| 142 | 565446 | tjp2a |  |  | 14491 | 0.070 | 0.0329 | No |
| 143 | 81580 | cldna |  |  | 14659 | 0.062 | 0.0280 | No |
| 144 | 100005752 | cgnb |  |  | 14801 | 0.056 | 0.0239 | No |
| 145 | 474349 | magi1b |  |  | 14805 | 0.055 | 0.0241 | No |
| 146 | 555454 | myh10 |  |  | 14890 | 0.051 | 0.0217 | No |
| 147 | 403050 | pard3ab |  |  | 15104 | 0.041 | 0.0152 | No |
| 148 | 571256 | wasa |  |  | 15521 | 0.021 | 0.0021 | No |
| 149 | 415204 | arpc3 |  |  | 15534 | 0.020 | 0.0019 | No |
| 150 | 554861 | actr2b |  |  | 15691 | 0.012 | -0.0030 | No |
| 151 | 100141341 | arhgap17b |  |  | 15694 | 0.012 | -0.0030 | No |
| 152 | 445164 | tuba8l3 |  |  | 15740 | 0.009 | -0.0044 | No |
| 153 | 101884148\_1 | LOC101884148 |  |  | 15965 | -0.000 | -0.0115 | No |
| 154 | 557209 | cldn18 |  |  | 16070 | -0.004 | -0.0147 | No |
| 155 | 103911473 | LOC103911473 |  |  | 16218 | -0.008 | -0.0193 | No |
| 156 | 406714 | rap1ab |  |  | 16378 | -0.013 | -0.0243 | No |
| 157 | 335232 | prkab1b |  |  | 16449 | -0.016 | -0.0264 | No |
| 158 | 321070 | rock2b |  |  | 16494 | -0.017 | -0.0277 | No |
| 159 | 101885491 | mpdz |  |  | 16613 | -0.021 | -0.0313 | No |
| 160 | 334086 | hspa4b |  |  | 16634 | -0.021 | -0.0318 | No |
| 161 | 406685 | ppp2r1ba |  |  | 16873 | -0.029 | -0.0392 | No |
| 162 | 445076 | prkacab |  |  | 17201 | -0.041 | -0.0493 | No |
| 163 | 327263 | ppp2r2ab |  |  | 17326 | -0.047 | -0.0530 | No |
| 164 | 561589 | ezrb |  |  | 17527 | -0.055 | -0.0590 | No |
| 165 | 553350 | cgnl1 |  |  | 17547 | -0.056 | -0.0593 | No |
| 166 | 81587 | cldnh |  |  | 17589 | -0.058 | -0.0603 | No |
| 167 | 559784 | amotl2b |  |  | 17599 | -0.058 | -0.0602 | No |
| 168 | 751760 | llgl1 |  |  | 17716 | -0.063 | -0.0636 | No |
| 169 | 436905 | prkab1a |  |  | 17733 | -0.064 | -0.0637 | No |
| 170 | 100334486 | map3k1 |  |  | 17841 | -0.069 | -0.0667 | No |
| 171 | 81586 | cldng |  |  | 17843 | -0.069 | -0.0663 | No |
| 172 | 100149273 | mapk9 |  |  | 17865 | -0.070 | -0.0666 | No |
| 173 | 415242 | rap1aa |  |  | 17893 | -0.071 | -0.0671 | No |
| 174 | 550537 | stk11 |  |  | 18181 | -0.084 | -0.0757 | No |
| 175 | 570858 | sympk |  |  | 18221 | -0.086 | -0.0764 | No |
| 176 | 100330246 | LOC100330246 |  |  | 18265 | -0.088 | -0.0773 | No |
| 177 | 406411 | rhoaa |  |  | 18330 | -0.091 | -0.0788 | No |
| 178 | 101883172 | LOC101883172 |  |  | 18491 | -0.098 | -0.0833 | No |
| 179 | 450006 | myl9a |  |  | 18511 | -0.099 | -0.0834 | No |
| 180 | 81582 | cldnc |  |  | 18581 | -0.102 | -0.0850 | No |
| 181 | 561431 | myh9b |  |  | 18683 | -0.107 | -0.0876 | No |
| 182 | 449548 | ppp2r1bb |  |  | 18698 | -0.108 | -0.0874 | No |
| 183 | 393885 | zgc:56064 |  |  | 18701 | -0.108 | -0.0869 | No |
| 184 | 334086\_3 | hspa4b |  |  | 18741 | -0.110 | -0.0875 | No |
| 185 | 474323 | rap2c |  |  | 18821 | -0.114 | -0.0893 | No |
| 186 | 117507 | prkci |  |  | 18879 | -0.117 | -0.0905 | No |
| 187 | 386966 | erbb2 |  |  | 18881 | -0.117 | -0.0899 | No |
| 188 | 327198 | micall2a |  |  | 18892 | -0.118 | -0.0895 | No |
| 189 | 556594 | LOC556594 |  |  | 18932 | -0.119 | -0.0901 | No |
| 190 | 100001644 | ppp2r2ba |  |  | 18973 | -0.120 | -0.0907 | No |
| 191 | 30678 | pcna |  |  | 18983 | -0.121 | -0.0903 | No |
| 192 | 327204 | rac1a |  |  | 19053 | -0.124 | -0.0918 | No |
| 193 | 558720 | marveld2a |  |  | 19131 | -0.128 | -0.0935 | No |
| 194 | 678619 | prkag3b |  |  | 19261 | -0.133 | -0.0968 | No |
| 195 | 326719 | myl12.1 |  |  | 19292 | -0.135 | -0.0970 | No |
| 196 | 570842 | si:ch73-15n15.3 |  |  | 19316 | -0.136 | -0.0970 | No |
| 197 | 554168 | myh11a |  |  | 19328 | -0.137 | -0.0966 | No |
| 198 | 100002327 | LOC100002327 |  |  | 19578 | -0.147 | -0.1036 | No |
| 199 | 100170795 | prkab2 |  |  | 19617 | -0.149 | -0.1040 | No |
| 200 | 564081\_1 | dlg3 |  |  | 19888 | -0.161 | -0.1116 | No |
| 201 | 333938 | myh9a |  |  | 20081 | -0.170 | -0.1168 | No |
| 202 | 494075 | oclnb |  |  | 20278 | -0.178 | -0.1220 | No |
| 203 | 567621 | tiam1a |  |  | 20311 | -0.180 | -0.1220 | No |
| 204 | 724019 | crb3b |  |  | 20399 | -0.183 | -0.1237 | No |
| 205 | 336839 | cdc42 |  |  | 20515 | -0.189 | -0.1263 | No |
| 206 | 337084 | myl12.2 |  |  | 20670 | -0.197 | -0.1300 | No |
| 207 | 793952 | si:dkey-253i9.4 |  |  | 20817 | -0.205 | -0.1335 | No |
| 208 | 569217 | jam3b |  |  | 20863 | -0.208 | -0.1338 | No |
| 209 | 568316 | prkag2b |  |  | 21087 | -0.220 | -0.1396 | No |
| 210 | 100333734 | LOC100333734 |  |  | 21172 | -0.225 | -0.1410 | No |
| 211 | 100005261 | jam2a |  |  | 21198 | -0.226 | -0.1405 | No |
| 212 | 436897 | cldn11a |  |  | 21316 | -0.231 | -0.1429 | No |
| 213 | 30222 | ccnd1 |  |  | 21522 | -0.242 | -0.1481 | No |
| 214 | 550254 | arhgap17a |  |  | 21535 | -0.243 | -0.1471 | No |
| 215 | 399482 | arpc5b |  |  | 21672 | -0.250 | -0.1500 | No |
| 216 | 101882925 | pard3aa |  |  | 22073 | -0.271 | -0.1611 | No |
| 217 | 573644 | cldn15a |  |  | 22108 | -0.273 | -0.1607 | No |
| 218 | 406303 | tuba2 |  |  | 22113 | -0.274 | -0.1592 | No |
| 219 | 100038776 | actr3b |  |  | 22285 | -0.283 | -0.1631 | No |
| 220 | 327272\_1 | slc9a3r1a |  |  | 22390 | -0.289 | -0.1647 | No |
| 221 | 553350\_1 | cgnl1 |  |  | 22514 | -0.297 | -0.1670 | No |
| 222 | 100005075 | prkcea |  |  | 22637 | -0.304 | -0.1691 | No |
| 223 | 406250 | actr3 |  |  | 22666 | -0.305 | -0.1683 | No |
| 224 | 559080 | cftr |  |  | 22825 | -0.315 | -0.1715 | No |
| 225 | 559080\_1 | cftr |  |  | 22866 | -0.317 | -0.1710 | No |
| 226 | 58126 | runx1 |  |  | 22962 | -0.324 | -0.1722 | No |
| 227 | 373105 | rab13 |  |  | 23129 | -0.336 | -0.1756 | No |
| 228 | 406659 | rhoac |  |  | 23154 | -0.338 | -0.1744 | No |
| 229 | 406493 | myl9b |  |  | 23170 | -0.339 | -0.1730 | No |
| 230 | 114446 | dlg1 |  |  | 23345 | -0.351 | -0.1765 | No |
| 231 | 795591 | si:ch211-114n24.6 |  |  | 23557 | -0.364 | -0.1812 | No |
| 232 | 81584 | cldne |  |  | 23721 | -0.375 | -0.1842 | No |
| 233 | 497638 | dlg2 |  |  | 23796 | -0.380 | -0.1844 | No |
| 234 | 563056 | hcls1 |  |  | 23829 | -0.382 | -0.1833 | No |
| 235 | 555737\_1 | prkcz |  |  | 23911 | -0.387 | -0.1837 | No |
| 236 | 323696\_1 | f11r.1 |  |  | 24012 | -0.393 | -0.1847 | No |
| 237 | 550431 | cldn19 |  |  | 24220 | -0.408 | -0.1889 | No |
| 238 | 436719 | cldn15lb |  |  | 24304 | -0.414 | -0.1892 | No |
| 239 | 101884148 | LOC101884148 |  |  | 24339 | -0.415 | -0.1880 | No |
| 240 | 553434\_1 | msnb |  |  | 24425 | -0.416 | -0.1883 | No |
| 241 | 327263\_1 | ppp2r2ab |  |  | 24575 | -0.420 | -0.1907 | No |
| 242 | 100006041 | rhoab |  |  | 24773 | -0.436 | -0.1945 | No |
| 243 | 403310 | cacna1da |  |  | 24910 | -0.442 | -0.1963 | No |
| 244 | 327068 | arpc2 |  |  | 25071 | -0.452 | -0.1988 | No |
| 245 | novel.818 | - |  |  | 25208 | -0.460 | -0.2005 | No |
| 246 | 101886083 | LOC101886083 |  |  | 25338 | -0.469 | -0.2020 | No |
| 247 | 555737 | prkcz |  |  | 25364 | -0.472 | -0.2001 | No |
| 248 | 57935 | actb2 |  |  | 25530 | -0.487 | -0.2026 | No |
| 249 | 557693 | rapgef2 |  |  | 25718 | -0.504 | -0.2057 | No |
| 250 | 550497 | zgc:110333 |  |  | 25754 | -0.507 | -0.2040 | No |
| 251 | 436612\_1 | cldn7a |  |  | 26021 | -0.531 | -0.2094 | No |
| 252 | 562525 | cldn2 |  |  | 26053 | -0.534 | -0.2074 | No |
| 253 | 100150331 | si:ch211-191a24.4 |  |  | 26192 | -0.547 | -0.2087 | No |
| 254 | 81583 | cldnd |  |  | 26560 | -0.584 | -0.2170 | No |
| 255 | 793952\_2 | si:dkey-253i9.4 |  |  | 26641 | -0.592 | -0.2162 | No |
| 256 | 561184 | nf2a |  |  | 26650 | -0.593 | -0.2132 | No |
| 257 | 415190 | zgc:86896 |  |  | 26776 | -0.607 | -0.2137 | No |
| 258 | 406440 | prkag1 |  |  | 26779 | -0.607 | -0.2104 | No |
| 259 | 100332055\_1 | prkceb |  |  | 26882 | -0.617 | -0.2101 | No |
| 260 | 114446\_2 | dlg1 |  |  | 27400 | -0.657 | -0.2228 | No |
| 261 | 555703\_1 | map3k5 |  |  | 27481 | -0.671 | -0.2216 | No |
| 262 | 563122 | marveld2b |  |  | 27574 | -0.682 | -0.2206 | No |
| 263 | 555703 | map3k5 |  |  | 27827 | -0.719 | -0.2246 | No |
| 264 | 81589 | cldnj |  |  | 28023 | -0.745 | -0.2266 | Yes |
| 265 | 556734 | itgb1a |  |  | 28051 | -0.750 | -0.2232 | Yes |
| 266 | 110438182 | LOC110438182 |  |  | 28145 | -0.763 | -0.2219 | Yes |
| 267 | 562838 | rac1b |  |  | 28146 | -0.763 | -0.2176 | Yes |
| 268 | 556226 | cdc42l2 |  |  | 28186 | -0.770 | -0.2145 | Yes |
| 269 | 777730 | cdk4 |  |  | 28245 | -0.777 | -0.2120 | Yes |
| 270 | 100005301 | jam2b |  |  | 28369 | -0.799 | -0.2114 | Yes |
| 271 | 564081 | dlg3 |  |  | 28531 | -0.827 | -0.2118 | Yes |
| 272 | 100006208 | LOC100006208 |  |  | 28738 | -0.866 | -0.2135 | Yes |
| 273 | 562264 | arhgef18b |  |  | 28807 | -0.880 | -0.2107 | Yes |
| 274 | 100002802 | ppp2r2ca |  |  | 28892 | -0.897 | -0.2083 | Yes |
| 275 | 561276\_1 | arhgef2 |  |  | 28912 | -0.902 | -0.2039 | Yes |
| 276 | 81592 | cldn11b |  |  | 28966 | -0.912 | -0.2004 | Yes |
| 277 | 100006166 | si:ch211-1f22.1 |  |  | 28986 | -0.917 | -0.1959 | Yes |
| 278 | 114456 | arpc4 |  |  | 29163 | -0.962 | -0.1961 | Yes |
| 279 | 573122 | tuba1c |  |  | 29368 | -1.020 | -0.1968 | Yes |
| 280 | 101884327 | LOC101884327 |  |  | 29590 | -1.090 | -0.1977 | Yes |
| 281 | 323696 | f11r.1 |  |  | 29807 | -1.166 | -0.1979 | Yes |
| 282 | 445305\_1 | arpc4l |  |  | 29885 | -1.197 | -0.1937 | Yes |
| 283 | 792699 | rac1l |  |  | 30004 | -1.247 | -0.1904 | Yes |
| 284 | 798632\_1 | myl6 |  |  | 30126 | -1.302 | -0.1869 | Yes |
| 285 | 559392 | ppp2r2cb |  |  | 30352 | -1.418 | -0.1861 | Yes |
| 286 | 100005566\_1 | f11r.2 |  |  | 30374 | -1.432 | -0.1787 | Yes |
| 287 | 797651 | jam3a |  |  | 30704 | -1.675 | -0.1797 | Yes |
| 288 | 101883269 | LOC101883269 |  |  | 30732 | -1.709 | -0.1710 | Yes |
| 289 | 60635\_1 | cldn7b |  |  | 30832 | -1.786 | -0.1641 | Yes |
| 290 | 556298 | micall2b |  |  | 30833 | -1.788 | -0.1540 | Yes |
| 291 | 57934 | actb1 |  |  | 31071 | -2.048 | -0.1500 | Yes |
| 292 | 100536545 | synpo |  |  | 31231 | -2.293 | -0.1422 | Yes |
| 293 | 110438642 | LOC110438642 |  |  | 31357 | -2.544 | -0.1319 | Yes |
| 294 | 81585 | cldnf |  |  | 31405 | -2.656 | -0.1184 | Yes |
| 295 | 569084\_1 | cldni |  |  | 31639 | -3.409 | -0.1067 | Yes |
| 296 | 286739\_1 | msna |  |  | 31674 | -3.584 | -0.0876 | Yes |
| 297 | 337226 | zgc:153867 |  |  | 31724 | -3.881 | -0.0674 | Yes |
| 298 | 792828 | pard6a |  |  | 31747 | -4.071 | -0.0452 | Yes |
| 299 | 436718\_1 | rhocb |  |  | 31770 | -4.295 | -0.0218 | Yes |
| 300 | 793158 | cdc42l |  |  | 31794 | -4.556 | 0.0030 | Yes |
Table: GSEA details [plain text format]

  

Fig 2: TIGHT\_JUNCTION(DRE04530)      
 Blue-Pink O' Gram in the Space of the Analyzed GeneSet

  

Fig 3: TIGHT\_JUNCTION(DRE04530): Random ES distribution      
 Gene set null distribution of ES for **TIGHT\_JUNCTION(DRE04530)**
